# Arterial Stiffness in Patients with Obstructive Sleep Apnea and Hypertension: A Narrative Review

**DOI:** 10.3390/medsci14040435

**Published:** 2026-07-25

**Authors:** Zorica Nestorović, Bojana Stojadinović, Aleksandra Bibić, Petar Videnović, Neriman Ezgin, Dragan Hrnčić

**Affiliations:** 1 Institute of Biophysics, Faculty of Medicine, University of Belgrade, 11000 Belgrade, Serbia; 2 Faculty of Medicine, University of Belgrade, 11000 Belgrade, Serbia; 3 Institute of Medical Physiology “Richard Burian”, Faculty of Medicine, University of Belgrade, 11000 Belgrade, Serbia; 4 Department of Biotechnology, Institute of Natural and Applied Sciences, Cukurova University, Adana 01330, Turkey

**Keywords:** obstructive sleep apnea, hypertension, arterial stiffness, pulse wave velocity, masked hypertension, resistant hypertension, nocturnal blood pressure

## Abstract

Arterial stiffness is a determinant of cardiovascular risk and has been increasingly associated with obstructive sleep apnea (OSA), although its independent contribution remains unclear due to the presence of comorbidities, the most important of which is hypertension. This narrative review synthesizes current evidence on the relationship between OSA, hypertension, and arterial stiffness, with a particular focus on the mediating role of hypertension and its clinical phenotypes. Evidence from observational and longitudinal studies indicates that OSA is generally associated with increased arterial stiffness, particularly in younger and normotensive individuals. A stepwise increase in pulse wave velocity (PWV) is consistently observed across control, OSA, hypertension, and combined OSA–hypertension groups, supporting an additive vascular burden. Hypertension emerges as a central correlate and amplifier of vascular remodeling, with its presence attenuating the independent association between OSA and arterial stiffness. Findings regarding masked hypertension are heterogeneous, with some studies showing increased PWV in OSA while others demonstrate minimal differences after adjustment. In contrast, resistant hypertension is consistently associated with high-risk hemodynamic and metabolic profiles, although the independent contribution of OSA to arterial stiffness in this group is often limited after multivariable adjustment. Large cohort evidence further suggests that associations between OSA and arterial stiffness surrogates are largely explained by age, blood pressure status, and cardiometabolic comorbidities. Because the evidence base is predominantly cross-sectional, these findings should be interpreted as showing that OSA’s association with arterial stiffening is closely intertwined with the presence and development of hypertension, rather than establishing a causal or temporal sequence. Future longitudinal and interventional studies are needed to clarify causal pathways and therapeutic implications.

## 1. Introduction

Arterial stiffness is a central determinant of vascular function and a well-established predictor of cardiovascular morbidity and mortality, including coronary artery disease, stroke, and all-cause mortality. Beyond its structural implications, arterial stiffness reflects a progressive loss of the hemodynamic buffering capacity of the arterial system. Under physiological conditions, elastic central arteries accommodate a substantial proportion of stroke volume during systole and maintain continuous blood flow during diastole through elastic recoil, commonly referred to as the Windkessel effect. This mechanism reduces systolic load and protects microcirculation from excessive pulsatile stress. With increasing arterial stiffness, this buffering capacity is impaired, resulting in increased pulse wave velocity (PWV), earlier return of reflected waves, and augmentation of central systolic pressure, contributing to endothelial dysfunction, impaired tissue perfusion, and target organ damage [1,2].

A strong and bidirectional relationship exists between arterial stiffness and hypertension, particularly in aging populations [3,4]. Elevated blood pressure promotes structural and functional changes in the arterial wall, while increased arterial stiffness contributes to systolic hypertension and widened pulse pressure. Central hemodynamic parameters, such as aortic pressure and pressure amplification, may better reflect vascular damage than peripheral measurements [5].

Obstructive sleep apnea (OSA) is a highly prevalent sleep-related breathing disorder characterized by recurrent upper airway obstruction, intermittent hypoxia, and sleep fragmentation, and is increasingly recognized as a major contributor to cardiovascular disease, independently associated with hypertension, coronary artery disease, heart failure, and stroke through intermittent hypoxia, sympathetic activation, oxidative stress, inflammation, and endothelial dysfunction [6,7]. The relationship between OSA and hypertension is particularly strong, with OSA highly prevalent among patients with resistant hypertension, masked hypertension, and abnormal nocturnal blood pressure patterns [8].

OSA may plausibly influence arterial stiffness both directly and indirectly via hypertension and abnormal blood pressure phenotypes, but the extent to which each pathway contributes remains unresolved. This uncertainty stems from three compounding sources of heterogeneity in the existing literature: (i) inconsistent definitions and severity thresholds for OSA across studies; (ii) variable classification of hypertension phenotypes (sustained, masked, resistant, nondipping); and (iii) the use of non-equivalent arterial stiffness measures (carotid–femoral PWV, brachial–ankle PWV, and pulse pressure), which differ in anatomical site and physiological meaning. Together, these inconsistencies limit direct comparison across studies and causal inference regarding OSA’s independent vascular contribution [9].

Two recent reviews have addressed adjacent aspects of this field but leave the present question open. Several prior reviews have comprehensively summarized the epidemiology, pathophysiological mechanisms, and clinical management of OSA-related hypertension and cardiovascular disease [10,11,12,13]. Doonan et al. [14] conducted a systematic review of 24 studies on OSA and arterial stiffness, establishing that OSA is broadly associated with increased stiffness. Filip et al. [15] subsequently added 13 further studies to Doonan’s review. However, these reviews predated most of the masked-, resistant-, and nondipping-hypertension literature synthesized here and did not examine hypertension phenotype as an explanatory factor. More recently, Shiina [16] reviewed the clinical management of OSA-related hypertension, focusing on antihypertensive and CPAP treatment strategies; arterial stiffness is discussed as one mechanistic feature among several, rather than as the primary outcome evaluated across hypertension phenotypes. Neither review therefore directly addresses whether the vascular effects attributed to OSA remain independent once blood pressure status and its clinical subtypes are accounted for—the specific gap the present review targets.

Accordingly, it is hypothesized that the association between OSA and arterial stiffness is largely intertwined with coexisting hypertension and its phenotypic variations, rather than representing a purely independent association.

Since considerable heterogeneity exists across published studies regarding diagnostic criteria for obstructive sleep apnea, definitions of hypertension and its phenotypes, methods used to assess arterial stiffness (including different pulse wave velocity modalities), study populations, and outcome measures, a narrative review approach was chosen. These methodological differences limit the feasibility and interpretability of quantitative pooling while supporting a narrative synthesis that critically evaluates the available evidence and identifies consistent patterns and knowledge gaps. Our aim was to map and critically integrate evidence across disparate hypertension phenotypes rather than to derive a pooled effect estimate.

A structured search was conducted in PubMed, Scopus, and Web of Science for studies published in English from January 2000 to March 2025, using combinations of the terms obstructive sleep apnea, arterial stiffness, pulse wave velocity, hypertension, masked hypertension, resistant hypertension, and nocturnal blood pressure. Eligible studies were original observational, longitudinal, or interventional studies in adults that reported an arterial stiffness measure (cfPWV, baPWV, or pulse pressure) stratified by OSA and/or hypertension status; studies restricted to pediatric populations, case reports, and non-English-language articles were excluded. Landmark earlier studies were additionally included by hand-search when they provided foundational evidence. Given the narrative design, no formal risk-of-bias scoring (e.g., GRADE, Newcastle–Ottawa) was applied; instead, study design, sample size, and major limitations were tabulated for each included study (Table 1) to allow the reader to weigh the strength of each finding.

## 2. OSA, Hypertension, and Arterial Stiffness

### 2.1. Early Evidence Linking OSA, Hypertension, and Arterial Stiffness

Drager et al. [17] demonstrated a stepwise increase in carotid–femoral pulse wave velocity (cfPWV) across control (8.7 ± 0.3 m/s), OSA-only (10.2 ± 0.2 m/s), hypertension-only (10.7 ± 0.3 m/s), and combined OSA plus hypertension groups (12.1 ± 0.4 m/s). Participants were carefully matched for age, sex, and BMI and were free of major cardiovascular comorbidities. Importantly, resistant hypertension was more prevalent in the combined group (33.3%) compared with hypertension-only patients (20%), supporting a synergistic vascular burden.

Similarly, Tsioufis et al. [18] confirmed significantly higher cfPWV in hypertensive patients with OSA compared with those without OSA (8.56 ± 0.49 vs. 7.85 ± 0.93 m/s, *p* = 0.001), with significance persisting after multivariable adjustment.

### 2.2. Masked Hypertension and Nocturnal Blood Pressure Phenotypes

Drager et al. [19] showed progressive increases in cfPWV from controls (8.7 ± 0.7 m/s) to OSA without masked hypertension (9.4 ± 1.0 m/s), reaching the highest levels in OSA with masked hypertension (10.6 ± 1.1 m/s). Masked hypertension prevalence was 30.2% in OSA patients versus 11.1% in controls. Multivariable analysis identified daytime systolic blood pressure and nocturnal hypoxemia as independent predictors of PWV.

In contrast, Baguet et al. [20] reported no significant difference between OSA patients with normotension (8.3 ± 0.2 m/s) and masked hypertension (8.2 ± 0.2 m/s), while sustained hypertension showed significantly higher stiffness (9.9 ± 0.3 m/s), indicating heterogeneity in masked hypertension phenotypes.

### 2.3. Sex-Specific Vascular Effects of OSA

Jenner et al. [21] demonstrated higher PWV in OSA patients in both sexes: men (12.7 ± 2.4 vs. 11.1 ± 2.2 m/s) and women (13.2 ± 2.2 vs. 11.8 ± 2.4 m/s). Nondipping blood pressure was significantly more frequent in OSA, particularly in women (65.2% vs. 41.4%). OSA remained independently associated with both PWV elevation and nondipping patterns in multivariable models.

### 2.4. Impact of Antihypertensive Therapy and Longitudinal Vascular Changes

Borges et al. [22] showed higher baseline PWV in OSA patients (10.3 ± 1.9 vs. 9.2 ± 1.7 m/s), but similar reductions in both groups after 18 months of antihypertensive therapy. Blood pressure control improved similarly in OSA and non-OSA groups, indicating that OSA did not blunt the vascular response to treatment. However, nocturnal diastolic BP reduction was greater in OSA patients.

### 2.5. Large Cohort Evidence and OSA Severity Effects

Kim et al. [23] demonstrated a dose–response relationship between OSA severity and baPWV in normotensive individuals (13.8 → 14.6 → 15.4 m/s), while no significant differences were observed in hypertensive participants (15.6–15.8 m/s). Over 6 years, arterial stiffness progression (ΔbaPWV) increased only in normotensive participants (0.81 → 0.85 → 1.55), consistent with a role for OSA in early vascular aging that precedes overt hypertension.

### 2.6. Pulse Pressure-Based Large Cohort Evidence

Saeed et al. [24] investigated the association between obstructive sleep apnea (OSA) and arterial stiffness in a large prospective Scandinavian cohort including 6408 individuals referred to suspected OSA. Respiratory polygraphy was used for OSA assessment (AHI > 15), and pulse pressure (PP) was used as a surrogate marker of arterial stiffness.

In unadjusted analyses, OSA was associated with a higher prevalence of elevated pulse pressure; however, this association was not significant after multivariable adjustment, with age, sex, and hypertension emerging as the main determinants. Subgroup analyses further showed no independent association between OSA and pulse pressure in normotensive, hypertensive, or treated hypertensive participants. The study indicates that OSA has a limited independent effect on pulse pressure, with observed associations largely driven by demographic and cardiometabolic factors.

### 2.7. Resistant Hypertension and High-Risk Populations

Roderjan et al. [25] showed higher cfPWV in OSA patients with resistant hypertension, particularly in true resistant hypertension (8.81 ± 1.86 vs. 8.28 ± 1.62 m/s). OSA was associated with nondipping BP (68.3%), nocturnal systolic hypertension (48.9%), diabetes (40.2%), and prior cardiovascular disease (36.9%). However, OSA was not independently associated with PWV in multivariable models, suggesting strong confounding by cardiometabolic clustering.

### 2.8. Summary of Key Patterns

Across studies, several consistent patterns emerge. OSA is associated with increased arterial stiffness in most populations, with the strongest effects observed in younger and normotensive individuals. Hypertension appears to amplify arterial stiffness, with the highest stiffness values observed in combined OSA–hypertension phenotypes. Masked and nocturnal hypertension are highly prevalent in OSA, although their association with arterial stiffness is heterogeneous. In resistant hypertension, OSA is strongly associated with adverse blood pressure phenotypes and comorbidity clustering, but its independent contribution to arterial stiffness is often attenuated. Longitudinal evidence further suggests that OSA may contribute to early vascular aging prior to the development of established hypertension.

## 3. Discussion

This narrative review provides a comprehensive synthesis of current evidence examining the relationship between obstructive sleep apnea (OSA), hypertension, and arterial stiffness. Arterial stiffness, most assessed by pulse wave velocity (PWV), is a robust and independent predictor of cardiovascular morbidity and mortality, including coronary artery disease, stroke, and all-cause mortality [26,27,28,29]. Beyond cardiovascular outcomes, increased arterial stiffness has also been associated with subclinical atherosclerosis, renal dysfunction, and microvascular damage [30,31,32].

Large-scale evidence further supports the prognostic value of arterial stiffness across populations. Aortic stiffness independently predicts cardiovascular and all-cause mortality in hypertensive patients [27], while meta-analytic data confirm its incremental value for cardiovascular event prediction beyond traditional risk factors [26,29]. Mechanistically, arterial stiffness reflects cumulative vascular injury, structural remodeling, and loss of arterial compliance, integrating both aging and disease-related processes [28].

### 3.1. OSA, Hypertension, and Arterial Stiffness: Core Evidence

Across heterogeneous populations, OSA is consistently associated with increased arterial stiffness, particularly when hypertension is present. Seminal studies demonstrated a stepwise increase in carotid–femoral PWV across control, OSA, hypertension, and combined OSA-hypertension groups, indicating an additive vascular burden [17]. Similarly, hypertensive patients with OSA exhibit significantly higher arterial stiffness compared with those without OSA, even after adjustment for confounders [18].

These findings align with broader cardiovascular evidence showing that OSA is strongly associated with systemic cardiovascular disease, including coronary artery disease, heart failure, and stroke [33,34,35]. The American Heart Association further recognizes OSA as an independent and modifiable cardiovascular risk factor [6]. Importantly, real-world clinical data also emphasize the underdiagnosis of OSA in high-risk cardiovascular populations [36].

### 3.2. Hypertension as a Central Correlate and Amplifier

A consistent finding across the literature is that hypertension is closely associated with, and appears to amplify, the relationship between OSA and arterial stiffness. OSA is highly prevalent in hypertensive populations and is particularly enriched in resistant hypertension phenotypes [37,38]. Furthermore, OSA is associated with both sustained and nocturnal blood pressure elevation, including non-dipping and masked hypertension patterns [21,39].

**Figure 1:**
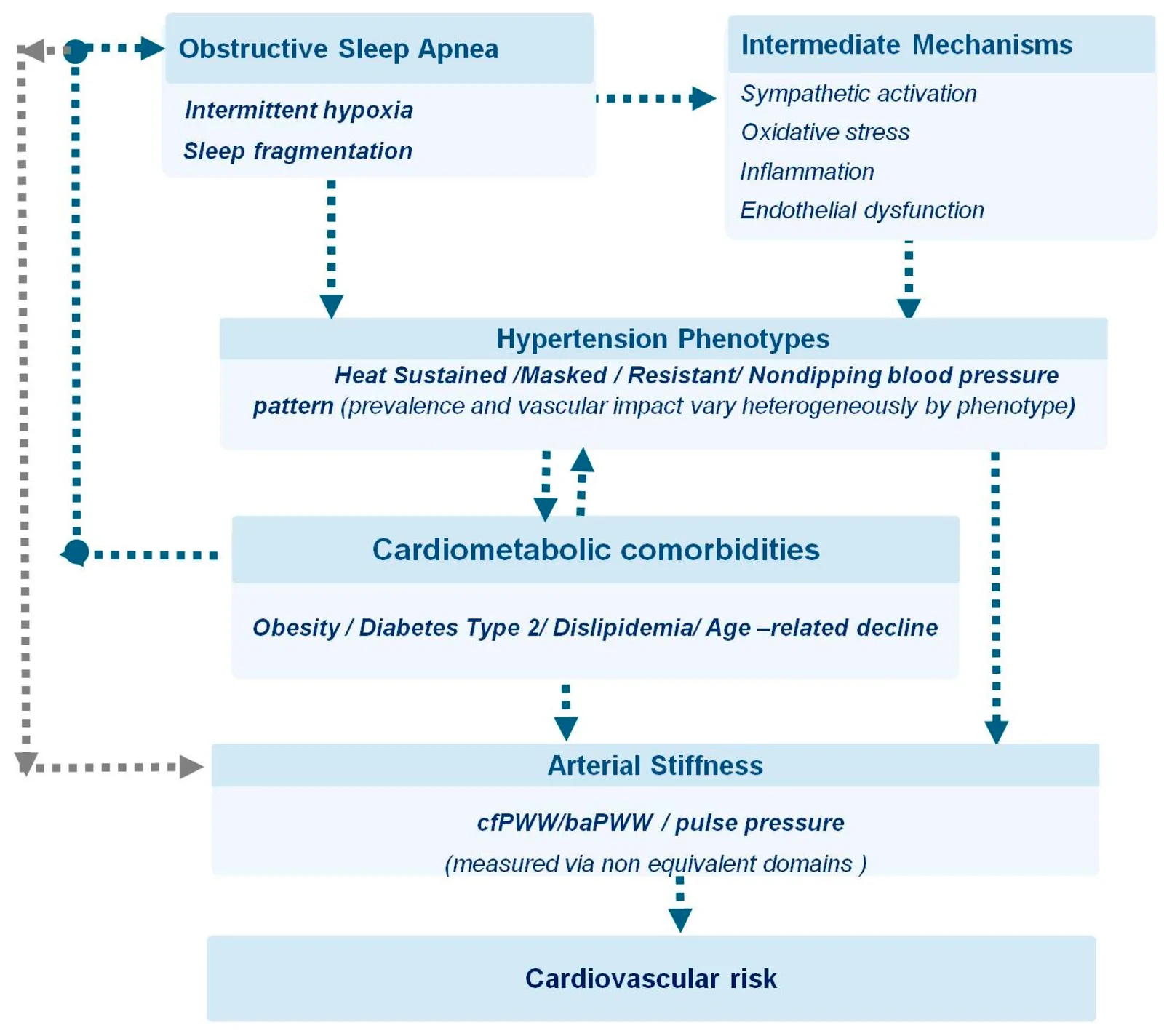
Conceptual pathway linking obstructive sleep apnea, hypertension phenotypes, and arterial stiffness. Obstructive sleep apnea (OSA) promotes intermittent hypoxia and sleep fragmentation, which trigger intermediate mechanisms—sympathetic activation, oxidative stress, systemic inflammation, and endothelial dysfunction—leading to the development of hypertension phenotypes, including sustained, masked, resistant, and nondipping blood pressure patterns. This hypertension-mediated route represents the principal pathway linking OSA to arterial stiffness supported across most reviewed studies. Cardiometabolic comorbidities (obesity, type 2 diabetes, dyslipidemia, age-related decline) cluster bidirectionally with hypertension phenotypes and independently contribute to arterial stiffening. A direct association between OSA and arterial stiffness is also observed in some studies but is consistently attenuated after multivariable adjustment for blood pressure status and comorbidities, indicating a secondary or residual rather than dominant contribution. Arterial stiffness itself is assessed through non-equivalent measurement domains—carotid–femoral pulse wave velocity (cfPWV, the central/aortic reference standard), brachial–ankle pulse wave velocity (baPWV, a composite central–peripheral measure), and pulse pressure (a simpler, load-dependent surrogate)—whose directionality of findings converges across studies even though absolute magnitudes are not directly comparable. Downstream, increased arterial stiffness contributes to cardiovascular risk, including coronary artery disease, stroke, and mortality.

The relationship between arterial stiffness and hypertension is bidirectional. Increased stiffness promotes systolic hypertension through wave reflection mechanisms, while chronic hypertension accelerates vascular remodeling and further stiffening [4,27]. This feedback loop amplifies cardiovascular risk over time. For schematic illustration, see Figure 1.

However, evidence regarding masked hypertension remains heterogeneous. Some studies demonstrate higher PWV in OSA patients with masked hypertension [19], while others show minimal differences compared to normotensive individuals [20]. These discrepancies suggest that sustained hypertension may be a stronger determinant of structural vascular changes than early or masked phenotypes.

This interpretation is further supported by large cohort data showing a minimal independent association between OSA and pulse pressure after multivariable adjustment, with age and hypertension emerging as the dominant determinants of arterial stiffness surrogates [24].

These observations may, at least in part, be explained by sex-related hormonal influences on cardiovascular regulation. Estrogen exerts vasoprotective effects by enhancing endothelial nitric oxide production, reducing oxidative stress, and attenuating sympathetic nervous system activity. Consequently, the decline in estrogen levels after menopause may contribute to increased arterial stiffness and a higher susceptibility to hypertension in women with OSA [40,41]. Experimental and clinical evidence further suggests that sex hormones modulate neurovascular control and sympathetic activation, potentially contributing to sex-specific differences in vascular remodeling and blood pressure regulation in OSA [42,43]. These findings indicate that menopausal status and sex-specific physiological mechanisms should be considered when evaluating vascular risk in patients with OSA.

### 3.3. Resistant Hypertension and High-Risk Phenotypes

Resistant hypertension represents one of the most clinically relevant phenotypes in OSA. OSA prevalence in this population can reach extremely high levels, and it is frequently associated with nondipping blood pressure patterns and metabolic comorbidities [37,38]. In this context, OSA is associated with adverse hemodynamic profiles, although its independent effect on arterial stiffness may be attenuated after adjustment for comorbidities [25].

This supports the concept that arterial stiffness in resistant hypertension is driven by a multifactorial clustering of risk factors, including ageing, obesity, diabetes, dyslipidemia, and chronic sympathetic activation, rather than OSA alone [36].

### 3.4. Pathophysiological Mechanisms

The association between OSA and arterial stiffness is biologically plausible and supported by multiple converging mechanisms. Intermittent hypoxia induces sympathetic nervous system overactivity, oxidative stress, and systemic inflammation, all of which contribute to endothelial dysfunction [17,44]. Reduced nitric oxide bioavailability further impairs vascular relaxation and promotes arterial remodeling.

Repeated hypoxia–reoxygenation cycles also trigger metabolic dysregulation, including insulin resistance and type 2 diabetes, which further exacerbate vascular injury [34]. Additionally, OSA has been linked to early atherosclerotic changes and vascular remodeling even in preclinical stages [17,33].

**Figure 2:**
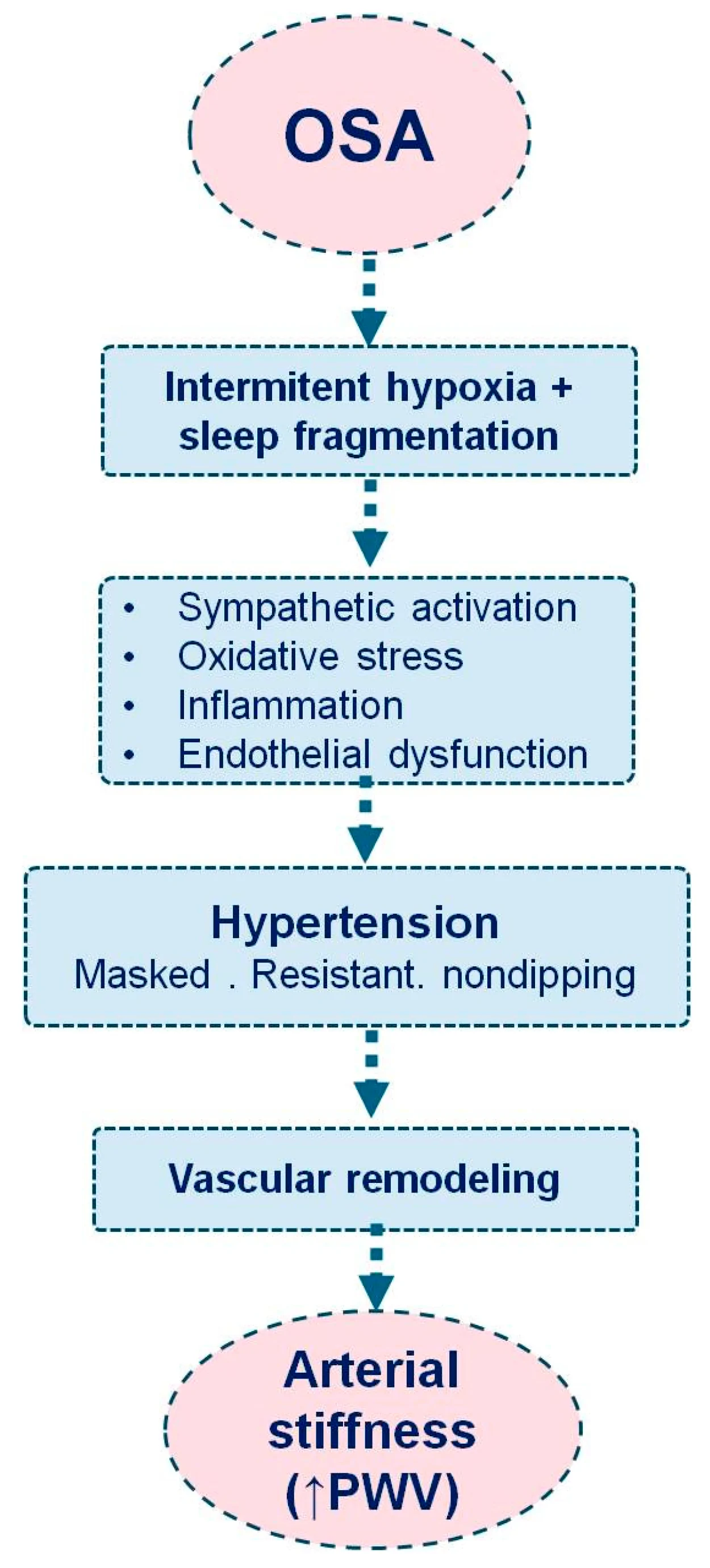
Proposed mechanisms linking obstructive sleep apnea, hypertension, and arterial stiffness. Obstructive sleep apnea (OSA) promotes intermittent hypoxia and sleep fragmentation, leading to sympathetic nervous system activation, oxidative stress, systemic inflammation, and endothelial dysfunction. These mechanisms contribute to the development of hypertension, including masked hypertension, resistant hypertension, and nondipping blood pressure patterns, which accelerate vascular remodeling and increase arterial stiffness, commonly assessed by pulse wave velocity (PWV). Although OSA may also exert direct vascular effects, current evidence suggests that hypertension is the principal correlate amplifying arterial stiffening and subsequent cardiovascular risk.

Cardiac structural adaptations, such as left ventricular hypertrophy, have been observed independently of hypertension, suggesting direct cardiac effects of OSA beyond blood pressure elevation [45]. These mechanisms reinforce OSA as a systemic cardiometabolic disorder rather than an isolated sleep condition [6,35]. For a conceptual framework, see Figure 2.

### 3.5. Longitudinal Evidence and Treatment Effects

Longitudinal data suggest that OSA contributes to early vascular aging. Severity of OSA is associated with progression of arterial stiffness in normotensive individuals, consistent with a role in early vascular remodeling prior to hypertension development [23].

Antihypertensive treatment appears to reduce arterial stiffness similarly in patients with and without OSA, suggesting that blood pressure control may partially mitigate OSA-related vascular damage [22]. For instance, study [23] confirmed over six years of follow-up that antihypertensive therapy has a beneficial effect, consistently demonstrating that it reduces the additional impact of hypertension on arterial stiffness in patients with hypertension. Nevertheless, the long-term impact of continuous positive airway pressure (CPAP) therapy on vascular remodeling remains a matter of ongoing investigation.

Evidence regarding the effects of continuous positive airway pressure (CPAP) on arterial stiffness has expanded considerably, although findings remain heterogeneous. In a meta-analysis, Vlachantoni et al. [46] demonstrated that CPAP therapy was associated with a significant reduction in arterial stiffness, particularly as assessed by carotid–femoral pulse wave velocity (cfPWV), suggesting that effective treatment of OSA may partially reverse vascular dysfunction. However, the magnitude of improvement varied across studies, reflecting differences in patient characteristics, treatment duration, and CPAP adherence. Similarly, Ning et al. [47] reported in a meta-analysis of randomized controlled trials that CPAP improved several cardiovascular biomarkers, including endothelial function and indices of arterial stiffness, with more pronounced benefits observed in patients with severe OSA and good treatment adherence. Restricting their analysis to randomized clinical trials, Chalegre et al. [48] confirmed a modest but significant reduction in PWV following CPAP therapy, whereas changes in augmentation index were inconsistent, suggesting that different measures of vascular function may respond differently to treatment. Furthermore, Jain et al. [49] demonstrated that the greatest improvements in central hemodynamic parameters, including central systolic blood pressure and pulse pressure, were achieved when CPAP was combined with weight loss, emphasizing the importance of comprehensive cardiometabolic risk management rather than CPAP therapy alone. Finally, Marcon et al. [50] concluded that although randomized trials generally support a favorable effect of CPAP on arterial stiffness, the available evidence is limited by relatively short follow-up periods, small sample sizes, and methodological heterogeneity, precluding definitive conclusions regarding the long-term reversal of vascular remodeling. Overall, short-term studies generally demonstrate modest improvements in vascular function following CPAP therapy, whereas robust evidence supporting sustained long-term reversal of arterial stiffness remains limited. Several factors may account for the limited and inconsistent vascular response to CPAP therapy despite its established effect on reducing sympathetic overactivity and intermittent hypoxia. First, the relationship between CPAP adherence and vascular benefit appears to be parameter-specific. Boneberg et al. [51] demonstrated that certain metabolic benefits of CPAP, such as improved insulin sensitivity, are only realized with supervised, high-intensity nightly use (≥8 h) and not with shorter usage (~4 h), supporting a dose-dependent relationship between treatment exposure and metabolic recovery. However, it is worth noting that Vlachantoni et al. [46]’s meta-analysis of arterial stiffness outcomes, discussed above, found that neither the proportion of compliant patients nor the duration of CPAP use significantly moderated the magnitude of arterial stiffness reduction—indicating that while CPAP’s overall effect on PWV was robust, the dose–response relationship observed for metabolic parameters may not directly extend to structural vascular measures. This dissociation suggests that CPAP-related metabolic and vascular recovery may follow distinct time courses, with structural arterial changes potentially requiring either longer treatment exposure [52] or independent management of coexisting hypertension and metabolic risk factors to achieve measurable improvement. Collectively, these findings reinforce the concept that hypertension—rather than OSA per se—remains the principal correlate of vascular injury, with CPAP serving as a necessary but insufficient component of comprehensive cardiometabolic risk management.

### 3.6. Synthesis of Evidence

Overall, the evidence converges on several key points:

- OSA is consistently associated with increased arterial stiffness [17,18]
- The effect is strongest in early disease and normotensive individuals [23]
- Hypertension acts as a major correlate and amplifier of vascular injury [4,27]
- Resistant hypertension and nocturnal blood pressure abnormalities represent high-risk phenotypes [37,39]
- In advanced cardiovascular disease, the independent contribution of OSA becomes less pronounced [25]

These findings support a model in which OSA interacts with hypertension and metabolic disease within a broader cardiometabolic network rather than acting as an isolated risk factor [6,36].

### 3.7. Clinical Implications

From a clinical perspective, patients with OSA, particularly those with resistant hypertension, masked hypertension, or nondipping blood pressure profiles, represent a high-risk vascular phenotype. Given the strong prognostic value of arterial stiffness, PWV assessment may provide additional risk stratification in these populations [29].

Optimal management should therefore extend beyond CPAP therapy and include aggressive control of blood pressure and metabolic risk factors. A multidisciplinary approach is essential for reducing long-term cardiovascular risk.

Arterial stiffness should be viewed not only as a consequence of hypertension and obstructive sleep apnea, but also as an intermediate vascular phenotype that may precede overt hypertension and contribute to cardiovascular risk. Early identification of increased arterial stiffness may therefore offer an opportunity for timely intervention before irreversible vascular remodeling develops.

Taken together, these findings support a hierarchical model in which OSA’s association with arterial stiffening is closely intertwined with the development of hypertension and associated cardiometabolic abnormalities, rather than reflecting OSA as an independent determinant of vascular remodeling.

### 3.8. Methodological Considerations

An additional methodological consideration is the heterogeneity of arterial stiffness assessment across the included studies. Carotid–femoral pulse wave velocity (cfPWV), the reference standard for central arterial stiffness, is measured over the aortic pathway and best reflects central elastic-artery properties directly relevant to left ventricular afterload. Brachial–ankle PWV (baPWV), used in Kim et al. [23], traverses both central elastic and peripheral muscular arterial segments and is additionally influenced by peripheral vascular tone and blood pressure at the time of measurement; it therefore captures a composite of central and peripheral stiffness rather than a pure central measure. Consequently, absolute PWV values obtained using different methodologies should not be directly compared. Pulse pressure, used by Saeed et al. [24], is the simplest surrogate but is heart-rate- and stroke-volume-dependent and does not isolate arterial wall stiffness from other hemodynamic determinants. Because these three measures index overlapping but non-identical physiological constructs, the studies summarized in Table 1 should be regarded as convergent rather than directly pooled evidence: consistent directionality across measures strengthens confidence in the overall association, but the magnitude of effect sizes is not directly comparable across studies using different techniques. Therefore, direct comparisons across studies should be interpreted with caution, and future research would benefit from greater methodological standardization, ideally converging on cfPWV as the reference measure where feasible. No formal risk-of-bias or quality assessment of reported studies in Table 1 was performed herein since this is a narrative review.

A further limitation of the evidence base itself, distinct from measurement heterogeneity, is that it is overwhelmingly cross-sectional (Table 1); only Borges et al. [22] and Kim et al. [23] provide longitudinal data. Cross-sectional designs cannot distinguish whether hypertension precedes, follows, or develops in parallel with OSA-related vascular change, and the “mediation” framing used descriptively throughout this review should be understood as a conceptual model derived from consistent cross-sectional patterns rather than a statistically tested mediation analysis. This is discussed further as a direction for future research below.

### 3.9. Key Messages

- OSA is associated with increased arterial stiffness, particularly in early disease stages.
- Hypertension is the principal correlate linking OSA with vascular remodeling.
- Masked, resistant, and nondipping hypertension represent high-risk vascular phenotypes.
- CPAP may improve vascular function; however, optimal vascular protection requires comprehensive management of hypertension and cardiometabolic risk factors.
- PWV assessment may improve cardiovascular risk stratification in selected patients with OSA.

## 4. Conclusions

In conclusion, OSA is consistently associated with increased arterial stiffness, particularly in early disease stages and in the absence of advanced cardiovascular comorbidities. Hypertension emerges as a central correlate and amplifier of arterial stiffening—closely linked to its development and intensifying its progression—thereby attenuating the apparent independent vascular association of OSA in high-risk populations, consistent with the hypothesis proposed in the Introduction. Because the current evidence base is predominantly cross-sectional, these conclusions describe a consistent pattern of association rather than an established causal sequence. Future research should prioritize longitudinal and interventional studies—including formal mediation analyses—to clarify causal pathways and evaluate whether targeted treatment strategies can fully reverse OSA-related vascular dysfunction.

## Figures and Tables

**Table 1:** Arterial stiffness measurements in obstructive sleep apnea and hypertension populations: study characteristics and key findings supporting narrative review.

Study	Design	Population	*n*	Arterial Stiffness Measure	Key Finding	Major Limitations	Adjustment Variables
Drager et al. [17]	Cross-sectional	OSA + HT vs. controls	60	cfPWV	Additive increase in arterial stiffness	Cross-sectional design; relatively small sample size; cannot establish causality; participants without major cardiovascular comorbidities may limit generalizability	Age, BMI, blood pressure
Tsioufis et al. [18]	Cross-sectional	Hypertensive pts	99	cfPWV	OSA increases PWV in HT	Cross-sectional study; included only newly diagnosed hypertensive patients; relatively small sample size	Age, BMI, systolic blood pressure
Drager et al. [19]	Cross-sectional	OSA + MH vs. controls	61	cfPWV	OSA increases PWV, especially in patients with masked hypertension	Cross-sectional design; relatively small sample size	Daytime systolic blood pressure, nocturnal hypoxemia
Baguet et al. [20]	Cross-sectional	BP phenotypes	126	cfPWV	HT stronger determinant than OSA	Cross-sectional design; small subgroup of patients with masked hypertension; limited power to detect differences	Age, BMI, blood pressure
Jenner et al. [21]	Cross-sectional	HT + OSA	95	cfPWV	Interaction with phenotype and sex	Cross-sectional design; hypertensive population only; findings may not be generalizable to normotensive individuals	Age, sex, BMI, nondipping systolic blood pressure
Borges et al. [22]	Cross-sectional	HT population	94	cfPWV	BP control reduces OSA effect	Cross-sectional design; observational follow-up within treated hypertensive patients;	Age, sex, BMI, blood pressure treatment
Kim et al. [23]	Cohort (6-year)	General population	1921	baPWV	OSA accelerates vascular aging	baPWV rather than cfPWV (reflects both central and peripheral arterial stiffness)	Age, sex, BMI, blood pressure, comorbidities
Saeed et al. [24]	Population-based cohort	Large cohort	6408	Pulse pressure	Minimal PP difference	Pulse pressure used as an indirect surrogate of arterial stiffness instead of direct PWV measurement; observational design	Age, sex, hypertension status
Roderjan et al. [25]	Cross-sectional	Resistant HT	376	cfPWV	Effect attenuated by comorbidity	Cross-sectional design; resistant hypertension with multiple cardiometabolic comorbidities introduces substantial confounding; independent effect of OSA difficult to isolate	Age, sex, BMI, multiple comorbidities confound

baPWV = brachial–ankle pulse wave velocity; cfPWV = carotid–femoral pulse wave velocity; HT = hypertension; MH = masked hypertension; OSA = obstructive sleep apnea; PP = pulse pressure; PWV = pulse wave velocity; pts = patients.

## Data Availability

No new data were created or analyzed in this study. Data sharing is not applicable to this article.
